# Effects of *Enterococcus faecium* (R8a) on nonspecific immune gene expression, immunity and intestinal flora of giant tiger shrimp (*Penaeus monodon*)

**DOI:** 10.1038/s41598-024-52496-4

**Published:** 2024-01-21

**Authors:** Xueliang Sun, Zhenzhen Fang, Hong Yu, Honghao Zhao, Yang Wang, Falin Zhou, Lin Zhao, Jingfeng Sun, Yunchen Tian

**Affiliations:** 1 Tianjin University, Environment College, 135 Yaguan Road, Haihe Education Park, Tianjin, 3003506 China; 2 Tianjin Agricultural University, 22 Jinjing Road, Tianjin, 300384 China; 3 Tianjin Key Laboratory of Aqua-Ecology and Aquaculture, College of Fisheries, Tianjin Agricultural University, 22 Jinjing Road, Tianjin, 300384 China; 4 Institute of South China Sea Oceanography, Chinese Academy of Sciences, 164 Xingang West Road, Haizhu District, Guangzhou, 510301 China

**Keywords:** Biological techniques, Immunology, Microbiology, Molecular biology

## Abstract

In this study, *Penaeus monodon* were gave basic feed supplemented with three levels of *Enterococcus faecium*. Then, the expression of non-specific immunity-related genes, and the activities of total antioxidant capacity (T-AOC), superoxide dismutase (SOD), malondialdehyde (MDA), acid phosphatase (ACP), alkaline phosphatase (AKP), phenol oxidase (PO) were evaluated. Meanwhile, the disease resistance test and intestinal flora determination were conducted. The results showed that the MDA levels of 2% and 5% *E. faecium* groups were significantly lower than that of the control group (*P* < 0.05). While the SOD and T-AOC and ACP and AKP of experimental groups were significantly higher (*P* < 0.05), the PO of experimental groups were significantly lower than that of the control group (*P* < 0.05). In addition, the expressions of immunity-related genes (*tlr22*, *dorsal*, *lysozyme*, *crustin*, *imd*, and *relish*) in the 2% and 5% *E. faecalis* groups were significantly greater than those in the control group (*P* < 0.05). After *P. monodon* was challenged with *Vibrio parahaemolyticus* for 7 days, the average cumulative mortality of *P. monodon* in the 2% and 5% groups were significantly lower than that in the 0% group (*P* < 0.05). With the increase of feeding time, the number of effective OTUs in each group showed a downward trend. At the 14th d, Proteobacteria, Bacteroidetes and Firmicutes, the dominant flora in the intestinal tract of *P. monodon*. In summary, supplied with *E. faecium* could increase the expression of non-specific immunity-related genes, enhance the immune capacity of *P. monodon.*

## Introduction

Giant tiger shrimp (*Penaeus monodon*), commonly known as bamboo shrimp, king shrimp, and cow shrimp, is also well known for its strong survivability and extreme resistances to high temperature, hypoxia stress, variation of salinity and drought. Thus, it occupies an increasingly prominent position among farmed shrimp species in Asia^1^. But, as is known, the shrimp is also susceptible to viruses, which has become a growing public concern^2^. Other infectious bacterial diseases are also common in *P. monodon* farming^3^. The frequent use of antibiotics rendered drug resistance to pathogens. Furthermore, the residual antibiotics not only killed the beneficial microorganisms in water, but also caused secondary pollution and endogenous infections^4^. For obtaining high-quality products while maintaining a sustainable aquaculture, the immune vaccines, Chinese herbal medicines and microbial agents are now advocated to replace the effect of antibiotics^5–7^.

Among them, probiotics refer to microbial agents that, within a certain concentration range, can improve the health of aquatic animals and water quality. As a substitute for antibiotics, the application of probiotics has rapidly developed in recent years^8^. It could reduce the incidence of animal diseases, increase the utilization ratio of feed, and promote the growth^9–11^. In addition, the use of probiotics could also improve the immunity and reduce the environmental pollution caused by aquaculture activities^12^. Therefore, the use of probiotics to prevent and control diseases has gradually become the focus of research in the aquaculture.

Vibrio vulnificus is a serious and harmful bacterial disease prevalent in shrimp aquaculture that has the potential to affect several shrimp species. There is a wide variety of Vibrio species that cause this disease^13^. Several recent studies have utilised broad-spectrum probiotics consisting of Bacillus, Lactic acid bacteria and other bacteria for shrimp aquaculture^14–18^. Among them, Lactic acid bacteria (LAB) has received much attention as an attractive probiotic for fish and shellfish culture^19^. L. acidophilus are commonly used in aquaculture as a biological control to protect various shrimp species from Vibrio spp. Lactobacillus acidophilus 04 was observed to significantly reduce the CMR% of shrimp in water bath experiments with Vibrio harveyi, Vibrio vulnificus or Vibrio europaeus ^20^. In a previous study, the addition of Mushroomella pentospora BD6 (10^10^ CFU/kg diet) to the diet for 60 consecutive days significantly increased the immune indices, i.e., PO and lysozyme activity (LYZ) in Vanamey carp.The expression of the Propoxygenase I (proPO I) gene was also significantly increased^21^. A study on non-lactic acid bacteria showed that the addition of Saccharomyces cerevisiae V HC-2 and Enterococcus faecalis NRW-2 (1 × 10^7^ CFU/g) to the diet of Penaeus vannamei for four consecutive weeks increased the humoral immune response of the shrimp. In addition, the survival rate (SR%) of shrimp in Vibrio parahaemolyticus water baths was also improved ^22^. *Enterococcus faecium* (R8a) is one kind of probiotic bacteria that maintains the physiological activity of animal intestine. It belongs to the lactic acid bacteria and can ferment carbohydrates to produce lactic acid in the animal intestine. *E. faecium* is a common probiotic in the animal intestine, but also an anaerobes, and easy to adhere and colonize in the intestinal tract^23^. As previously reported, *Enterococcus* spp. have been used as potential probiotics in various fish species due to their good tolerances to acidic pH of stomach and bile salts, excellent adhesion to intestinal epithelial surface, as well as their immune modulation and antagonistic activity toward enteropathogens^24–26^. The study has shown that the *E. faecium* V6-112 was able to resist the different gastrointestinal conditions and showed a high antioxidant property^27^. There were more studies on*E. faecium* in animal husbandry and other industries, whereas it is limited in the aquaculture.We previously isolated An Enterococcus faecium strain R8a from the intestine of healthy crucian carp Carassius auratus, which showed potential probiotic properties including^28^. In this research, *E. faecium* (R8a) was added to feeding diets to investigate its effect on the expression of non-specific immune genes, the activity of enzymes, disease resistance, and intestinal flora of *P. monodon*. The results of this study would provide the scientific basis for the application of *E. faecium* in the culture of *P. monodon*.

## Results

### Effects of supplied *E. faecium *on antioxidant indexes in hepatopancreas of *P. monodon*

#### Effects of supplied *E. faecium *on SOD activity in hepatopancreas of *P. monodon*

**Figure 1:**
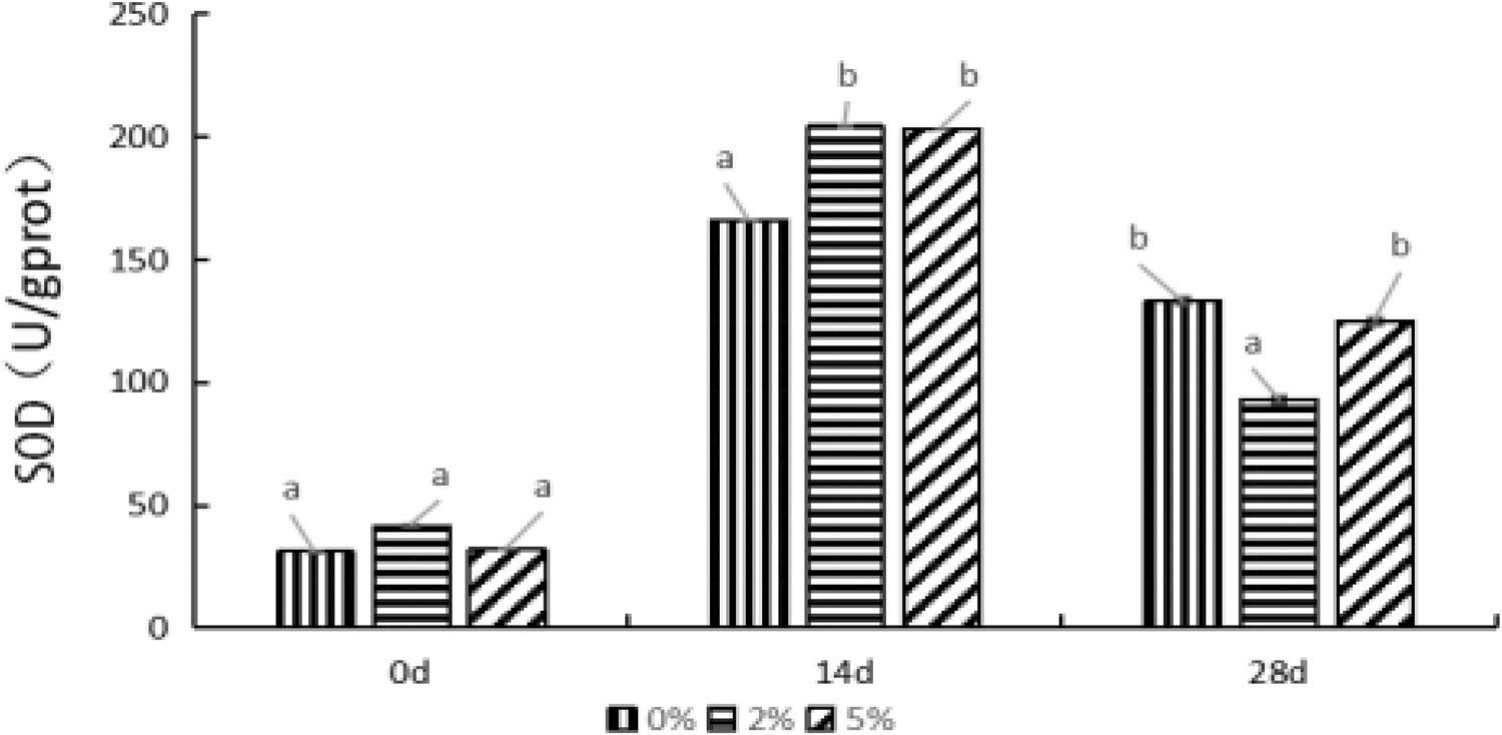
Effect of *E. faecium* on SOD activity in hepatopancreas of *P. monodon. Note*: Different letters represent significant differences.

As shown in Fig. 1, the SOD activity in *P. monodon* hepatopancreas from 0%, 2%, and 5% groups significantly increased after feeding for 14 d, compared with that of 0 d (*P* < 0.05). Meanwhile, the SOD activity in 2% and 5% groups were significantly higher than that of the control group (i.e. 0% group) (*P* < 0.05). After feeding for 28 d, the SOD activity in *P. monodon* hepatopancreas from 0%, 2%, and 5% groups significantly decreased relative to the results of 14 d (*P* < 0.05). At 28th day, the SOD activity in 2% group was significantly lower than that of the 0% and 5% groups (*P* < 0.05). The changes of SOD activity increased first, then decreased in each group with the increase of feeding time on the whole. It indicated that feeding *P. monodon* with *E. faecium* had a significant effect on SOD activity in hepatopancreas.

#### Effects of supplied *E. faecium *on MDA content in hepatopancreas of *P. monodon*

**Figure 2:**
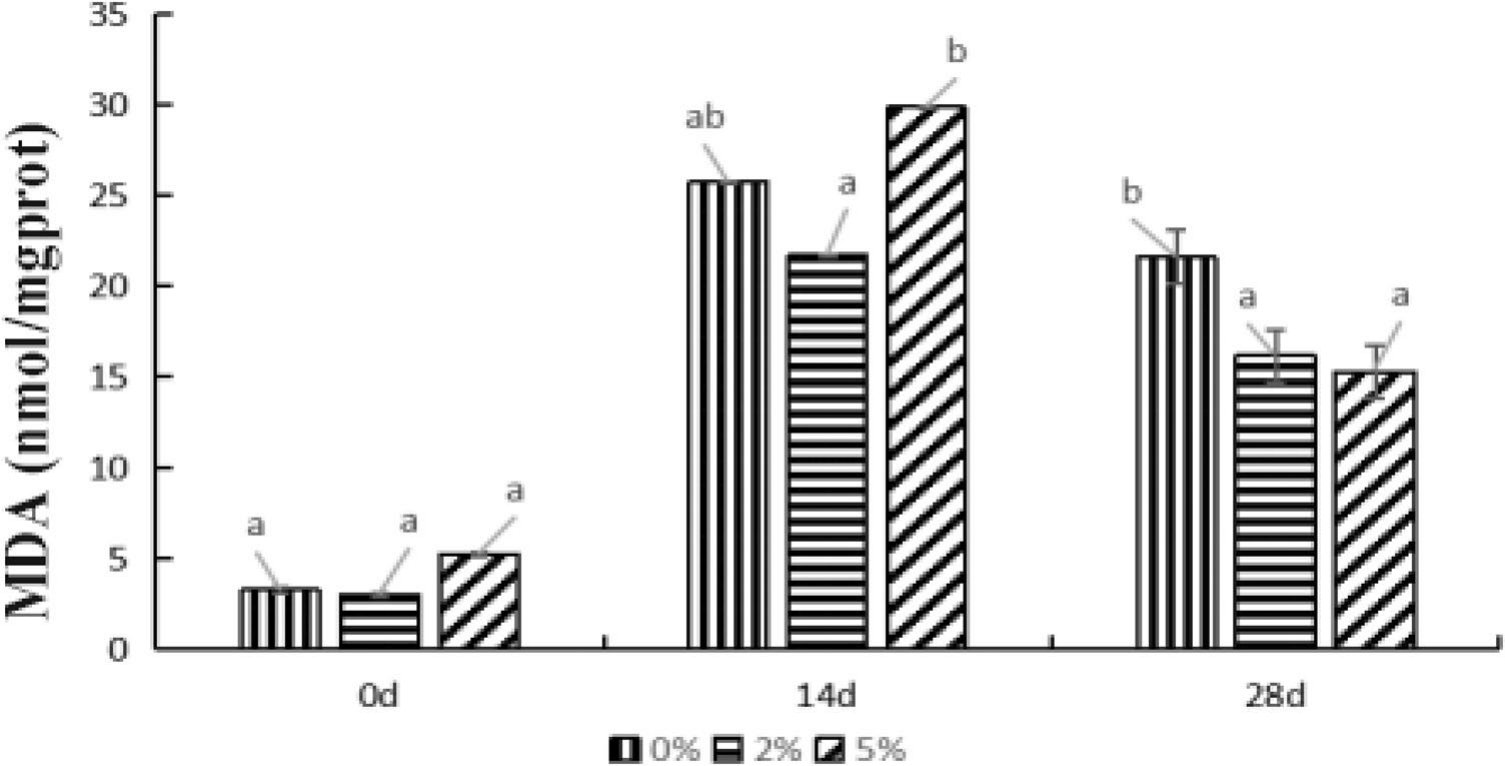
Effect of *E. faecium* on level of MDA in *P. monodon* hepatopancreas. *Note*: Different letters represent significant differences.

After feeding with levels of *E. faecium* for 14 d, the MDA content in *P. monodon* hepatopancreas from 0%, 2%, and 5% groups significantly increased when compared with the results of 0 d (*P* < 0.05) (Fig. 2). The MDA content exhibited no significant difference between the control and two experimental groups (including the 2% and 5% groups) (*P* > *0.05*), while MDA in 5% group was significantly greater than that in 2% group (*P* < *0.05*). When the feeding time extended to 28 d, the MDA level in 0% group was no significantly different from it on 14th day *(P* > *0.05)*, whereas significantly decreased in both 2% and 5% groups (*P* < *0.05*). Meanwhile, the level of MDA in 2% and 5% groups were significantly lower than that in the control group (*P* < *0.05*). Overall, the level of MDA showed a same trend with SOD activity as the feeding time going on. It indicated that feeding *E. faecium* had a significant effect on in MDA content the hepatopancreas of *P. monodon*.

#### Effects of supplied *E. faecium *on T-AOC activity in hepatopancreas of *P. monodon*

**Figure 3:**
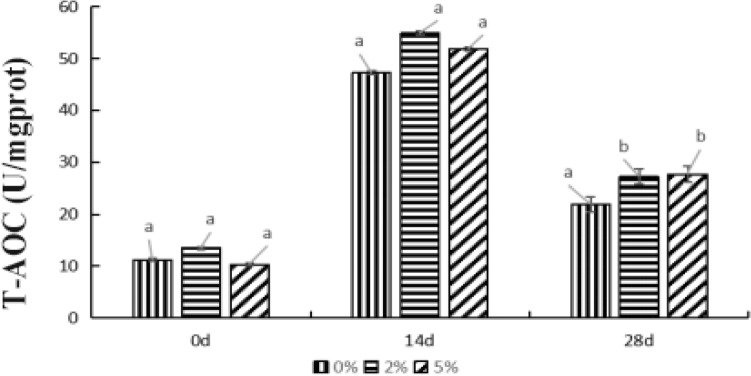
The effect of feeding *P. monodon* with feeds supplied with *E. faecium* on T-AOC in *P. monodon* hepatopancreas. *Note*: Different letters represent significant differences.

After feeding for 14 d, the T-AOC activity in 0%, 2%, and 5% groups’ samples significantly raised relative to the results of 0 d (*P* < *0.05*) (Fig. 3). T-AOC activity in the control group no differed from that in the two experimental groups. Until to the 28th day, the T-AOC activity in the 0%, 2%, and 5% groups all significantly declined (*P* < *0.05*), and the T-AOC activity in the two experimental groups were significantly higher than that in the control group (*P* < *0.05*). The above results indicated that the change of T-AOC activity during the feeding cycle was same as the changes of SOD and MDA in each group. It also means that feeding *E. faecium* had significant effect on the T-AOC activity in *P. monodon* hepatopancreas. Moreover, supplied *E. faecium* could significantly improve the antioxidant capacity of *P. monodon*.

### Effects of supplied *E. faecium *on non-specific immunity in hepatopancreas of *P. monodon*

#### Effects of levels of *E. faecium* on AKP activity in *P. monodon* hepatopancreas

**Figure 4:**
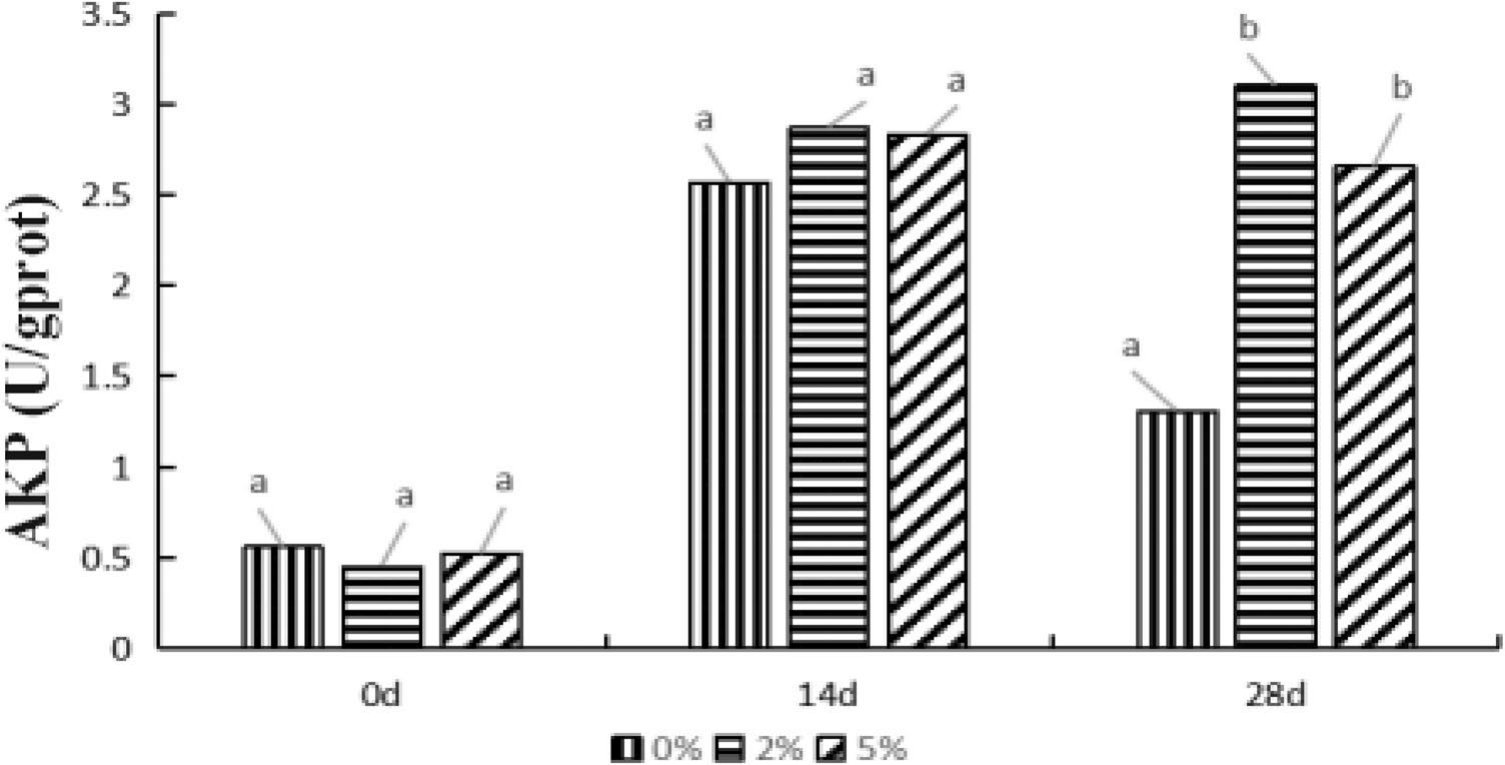
The alteration of AKP activity in *P. monodon* hepatopancreas with the changes of feeding time and levels of *E. faecium* in feeds. *Note*: Different letters represent significant differences.

The Fig. 4 revealed the change of AKP activity in the 0%, 2%, and 5% groups as the feeding time continues. The AKP activity in the three feeding groups significantly increased after 14 days compared to the 0 d (*P* < *0.05*). It showed no significant alteration in AKP activity between the 0% and experimental groups (*P* > *0.05*)*.* Until to the 28th day, the AKP activity in the 0% group significantly reduced relative to the 14th day (*P* < *0.05*), while significantly enhanced in the 2% and 5% groups. Whereas there was no significant difference observed in the comparison between 2 and 5% groups (*P* > *0.05*). In general, the activity of AKP increased in a time-dependent pattern, and the AKP values were the highest in the 2% group at the three test time points.

### Effects of levels of *E. faecium* on ACP activity in *P. monodon* hepatopancreas

**Figure 5:**
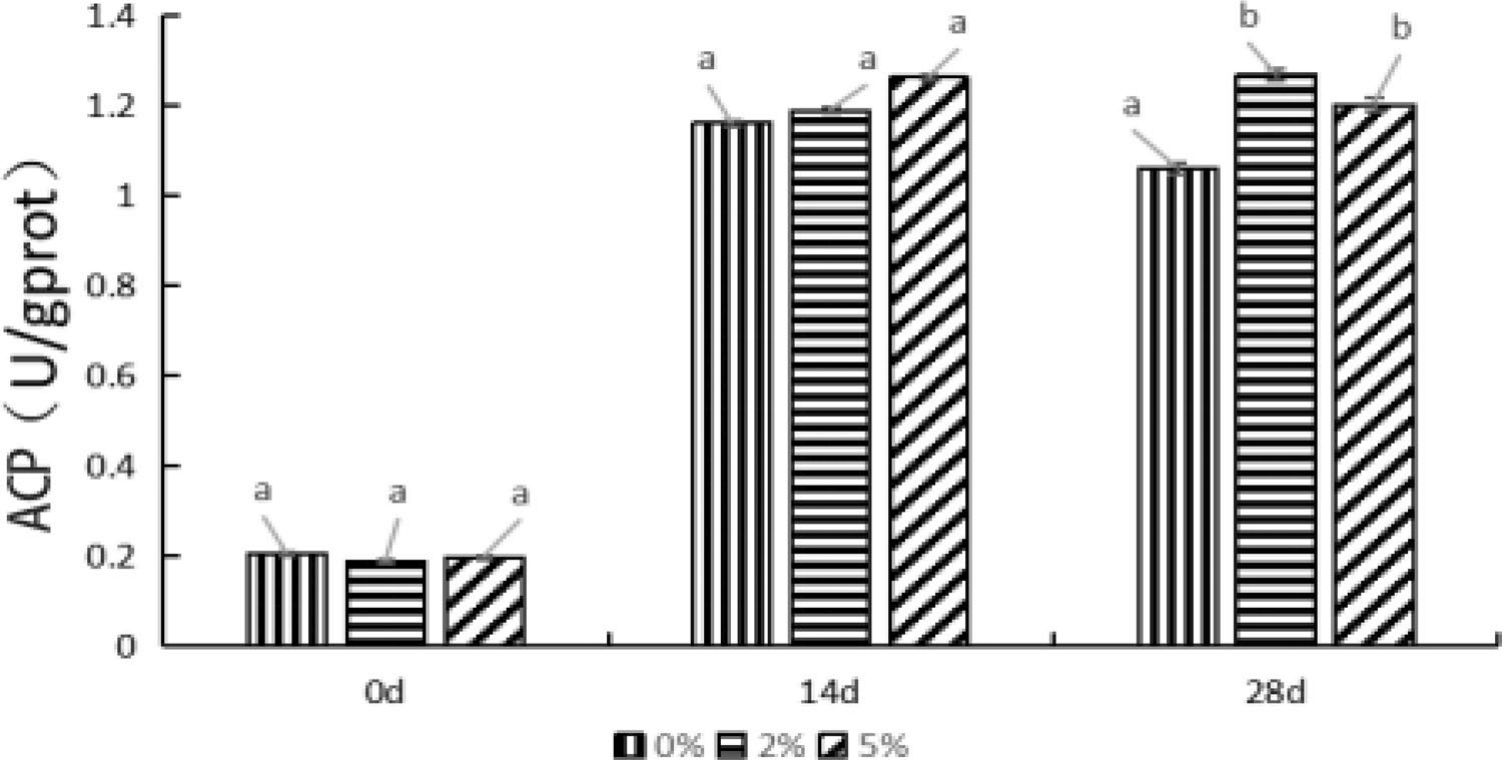
The influence of *E. faecium* supplement in feeds on ACP in *P. monodon* hepatopancreas. *Note*: Different letters represent significant differences.

As shown in Fig. 5, feeding *P. monodon* with levels of *E. faecium* had significant influence on ACP activity in hepatopancreas tissue. After feeding for 14 d, the ACP activity significantly increased compared with that of 0 d (*P* < *0.05*). Meanwhile, it showed no significant difference between the control and experimental groups (*P* > *0.05*). Until to the 28th day, the ACP activity of the control group significantly reduced relative to that of 14 d (*P* < *0.05*), and no remarkable difference was observed between the 2% and 5% groups (*P* > *0.05*). After feeding for 28 d, the ACP activity in the 2% and 5% groups were both significantly higher than that in the control group (*P* < *0.05*). Overall, except for the 2% group, the ACP activity increased with the feeding time, and the ACP activity of the other groups increased at first and then decreased, which illustrated that feeding *E. faecium* had a positive effect on the activity of ACP in a time-dependent pattern.

### Effects of *E. faecium* on PO activity in hepatopancreas of *P. monodon*

**Figure 6:**
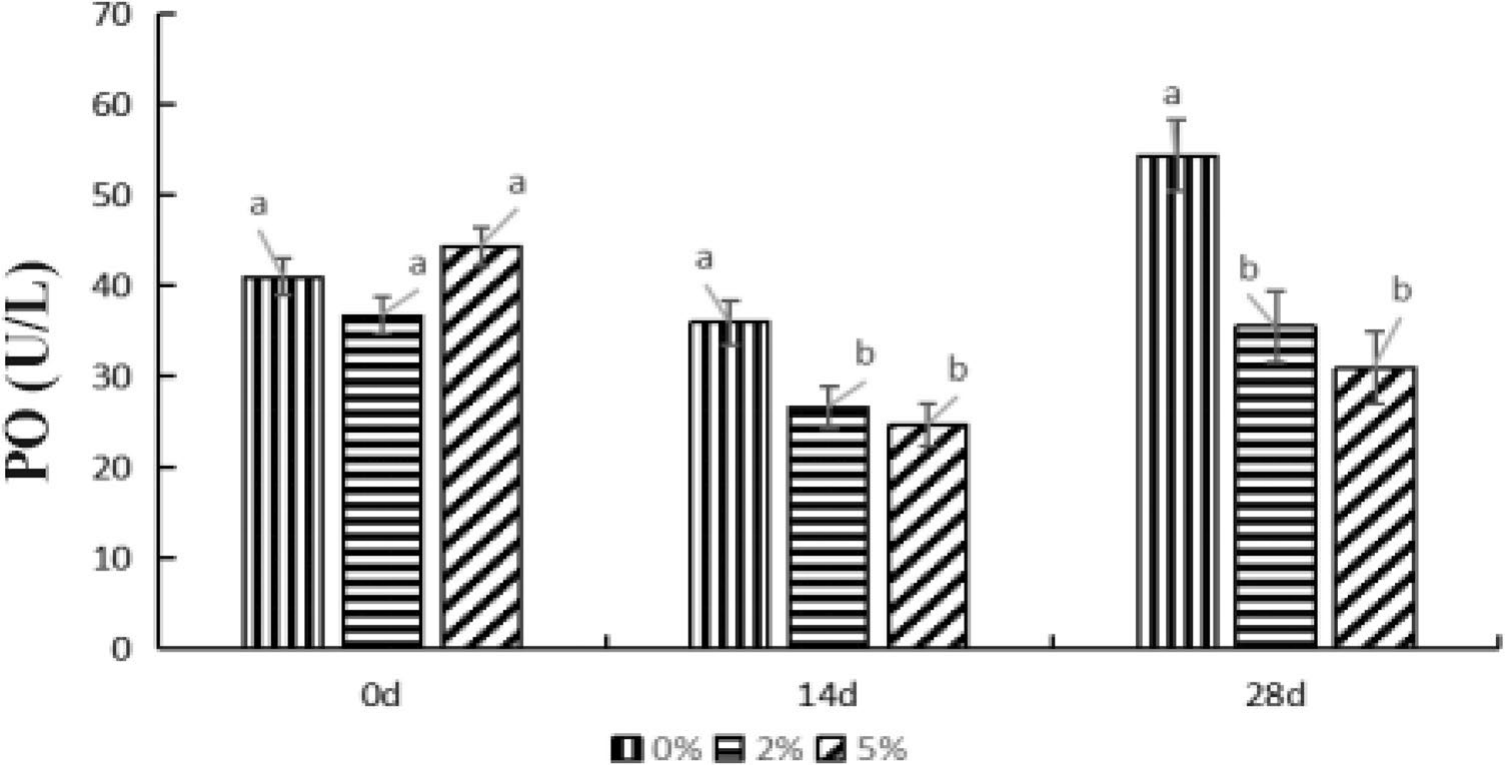
Effect of levels of *E. faecium* on PO in hepatopancreas of *P. monodon. Note*: Different letters represent significant differences.

The effects of various levels of *E. faecium* on PO activity in *P. monodon* hepatopancreas was visualized in Fig. 6. On day 14, the PO activity of 2% group’s samples was obviously lower than that of 0 d and 28 d (*P* < 0.05). On 28th day, the PO activity of control group further significantly increased compared to that of 0th and 14th day (*P* < 0.05). After feeding for 14 d and 28 d, the PO activity of the two experimental groups were both significantly lower than that of 0% group (*P* < 0.05). In general, *E. faecium* supplement in feeds had a significant effect on the PO activity of *P. monodon* hepatopancreas. As the feeding time continues, the PO activity of 2% and 5% groups decreased first, then enhanced.

### Effects of *E. faecium* on the expression levels of immunity-related genes in* P. monodon* hepatopancreas

**Figure 7:**
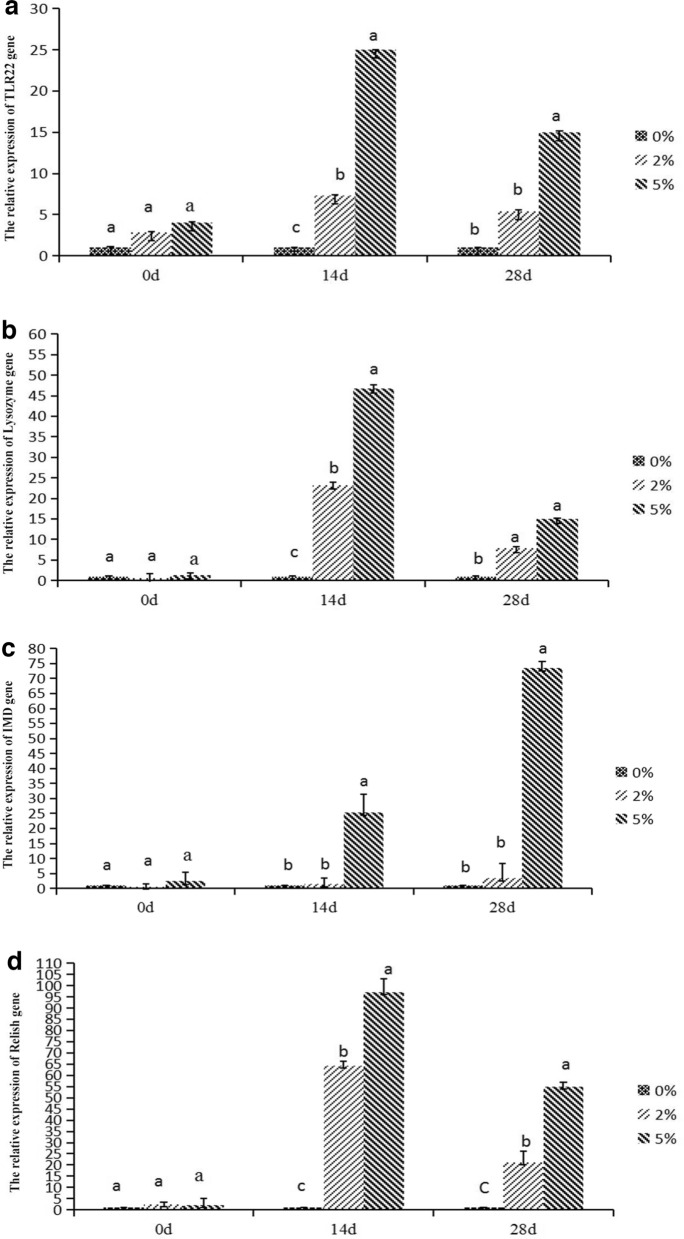

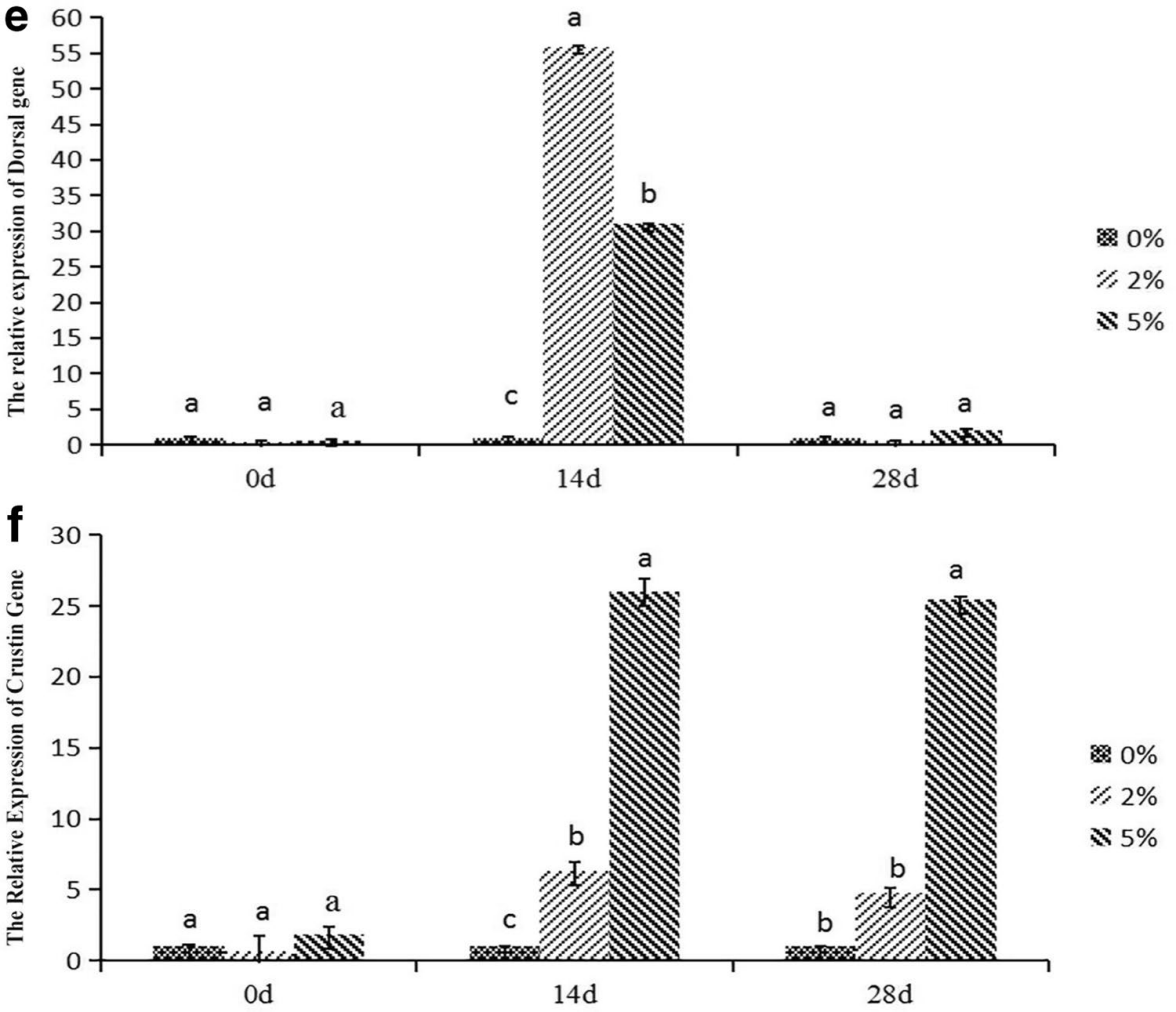
The influence of *E. faecium* supplement in feeds on expression of immune-related genes in *P. monodon* hepatopancreas. *Note*: Different letters represent significant differences.

The expressions of *tlr22*, *dorsal*, *lysozyme*, *crustin*, *imd*, and *relish* were down-regulated in all groups on 0th day of feeding, with no significant difference among groups (*P* < *0.05*) (Fig. 7). After feeding for 14 d, the maximum expression level of *Dorsal* was in 2% group, while the highest expressions of *TLR22*, *Lysozyme*, *Crustin*, *IMD*, *and Relish* were in 5% group, meanwhile, they were significantly different from those in the other groups (*P* < *0.05*). After feeding for 28 d, the expression levels of *TLR22*, *Dorsal*, *Lysozyme*, *Crustin*, *IMD*, and *Relish* were highest in the 5% group. Among them, the levels of *TLR22*, *Crustin*, *IMD*, and *Relish* were significantly different from those in the other groups (*P* < *0.05*). Taken together, feeding *P. monodon* with *E. faecium* had a significant effect on the expression of immune-related genes. Specifically, expressions of the genes in the experimental groups initially increased and then decreased in a time-dependent pattern.

### Challenge experiment

**Figure 8:**
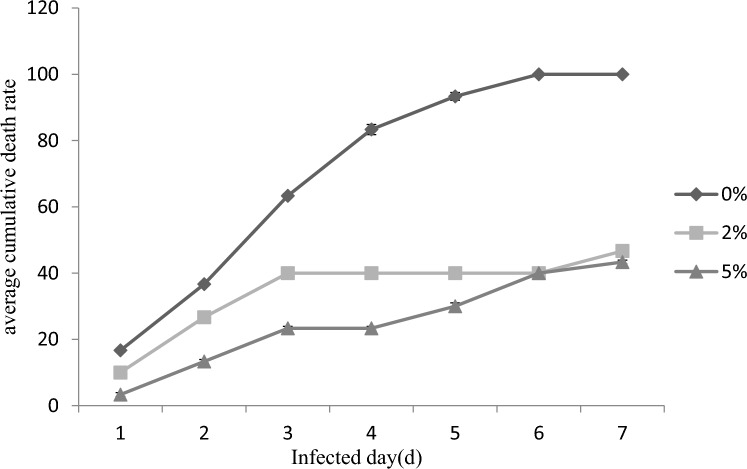
The effects of feeding *P. monodon* with feeding diets supplied with *E. faecium* on average cumulative death rate of *P. monodon.*

The average cumulative mortality of *P. monodon* after being challenged for 7 days was shown in Fig. 8. The average cumulative mortality of *P. monodon* was significantly lower in the 2% and 5% groups than that in the control group (*P* < 0.05). However, there was no significant difference between the 2% and 5% groups (*P* > 0.05). It means that the addition of *E. faecium* to the feeding diets could significantly reduce the cumulative mortality of *P. monodon*, and has a significant impact on the disease resistance of *P. monodon*.

### Analysis of the structure of intestinal flora of *P. monodon*

#### Effect of *E. faecium* on effective OTUs in intestine of *P. monodon*

**Figure 9:**
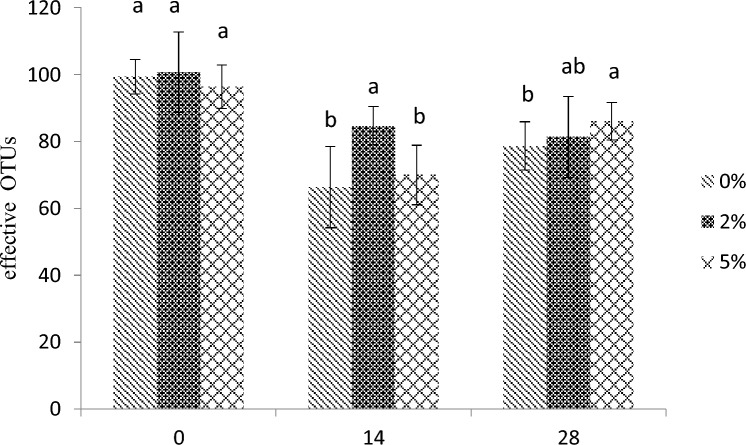
Effect of E. faecium on effective OTUs in intestine of *P. monodon*. *Note*: Different letters represent significant differences.

With the increase of breeding time, the number of effective OTUs in each group showed a downward trend. At 0d, there was no significant difference between the groups (*P* > 0.05). On the 14th day, the OTU of the 2% group was significantly higher than that of the 0% and 5% groups (*P* < 0.05), and there was no significant difference between the 0% and 5% groups (*P* > 0.05). At 28 days, the OTU of the 0% group was significantly lower than that of the 5% group (*P* < 0.05), and there was no significant difference between the 0% and 2% groups (*P* > 0.05) (Fig. 9). Overall, feeding *E. faecium* had a significant effect on the number of OTUs in the intestinal flora.

#### Effects of *E. faecium* on gut species diversity of* P. monodonon*

The coverage index (Coverage) was 1.00 in each group, indicating that the samples in each group met the sequencing requirements. At 0d and 14d, the richness Ace index of the 0% and 2% groups were higher than that of the 5% group, and The Ace value of the 2% group is the largest. The Chaol index of 5% group was lower than the 0% and 2% groups, and the 2% group is the largest. The other species diversity indices, Shannon's index (Shannon) and Simpson's index (Simpson), were not significantly different among the groups (Table 1). Overall, feeding *E. faecium* can improve the diversity and richness of intestinal flora.

**Table 1:** Effects of *Enterococcus faecium* on gut species diversity of *Penaeus monodonon* n = 3.

Breeding time (d)	Group (%)	ACE	Chao1	Simpson	Shannon	Coverage
0	0	122.55 ± 10.47	115.98 ± 7.37	0.13 ± 0.05	2.60 ± 0.29	1.00 ± 0.00
	2	123.25 ± 10.64	122.47 ± 20.96	0.11 ± 0.06	2.72 ± 0.41	1.00 ± 0.00
	5	113.97 ± 17.94	112.29 ± 11.99	0.21 ± 0.19	2.34 ± 0.76	1.00 ± 0.00
14	0	110.93 ± 10.11	105.06 ± 19.08	0.26 ± 0.02	1.66 ± 0.13	1.00 ± 0.01
	2	114.91 ± 39.27	115.61 ± 27.11	0.16 ± 0.02	2.24 ± 0.06	1.00 ± 0.00
	5	84.76 ± 4.88	86.83 ± 5.6	0.20 ± 0.06	1.95 ± 0.28	1.00 ± 0.00
28	0	119.81 ± 28.87	102.99 ± 13	0.27 ± 0.08	1.73 ± 0.28	1.00 ± 0.00
	2	117.30 ± 15.13	107.5 ± 10.33	0.23 ± 0.06	1.83 ± 0.27	1.00 ± 0.00
	5	109.60 ± 8.82	110.40 ± 6.96	0.22 ± 0.02	2.08 ± 0.03	1.00 ± 0.00

#### Communities and relative content of bacteria at the level of Phylum in intestine of *P. monodon*

**Figure 10:**
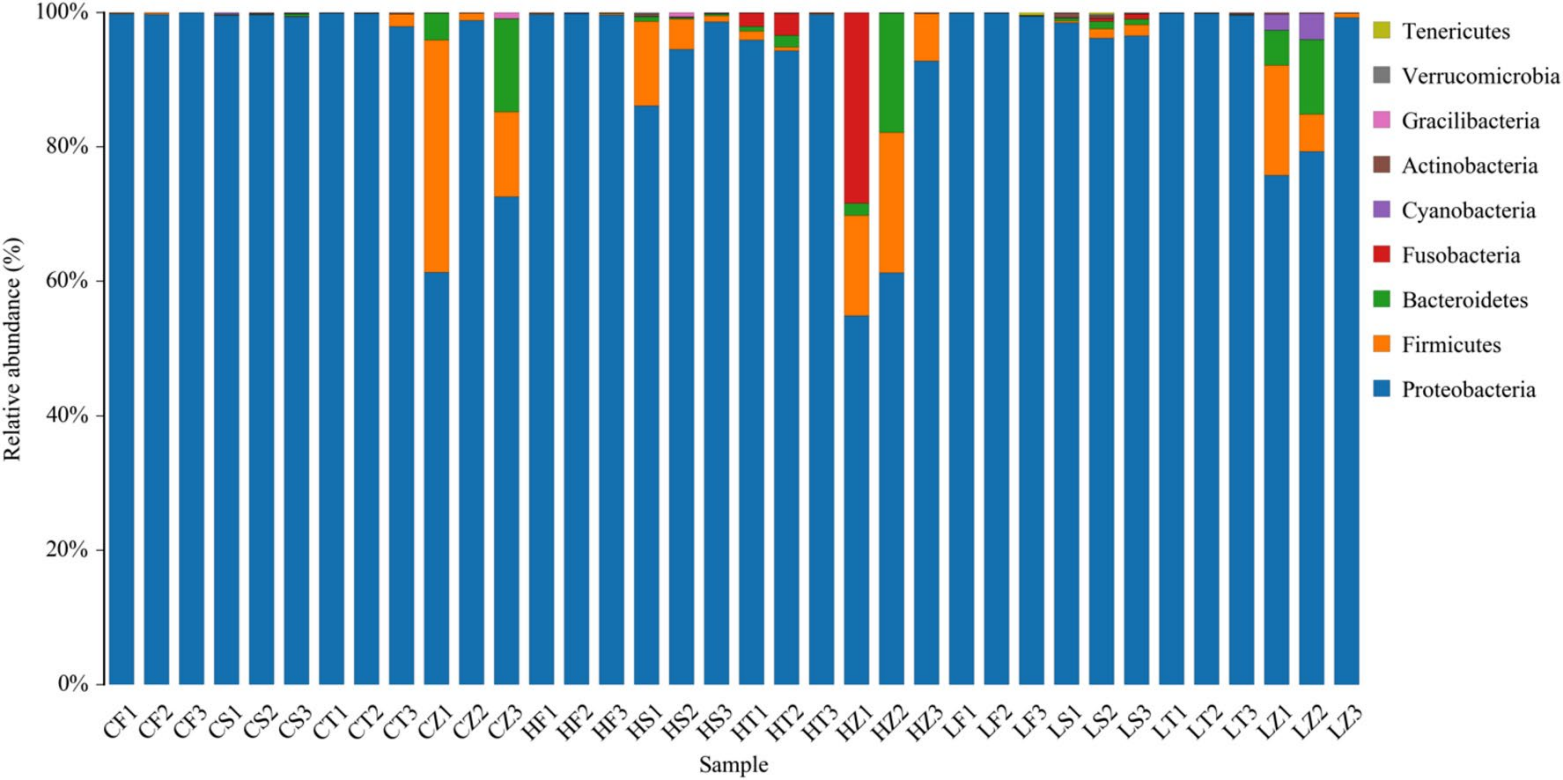
Communities and relative content of bacteria at the level of Phylum in intestine of *P. monodon.*

In this experiment, the intestinal flora of monodon was mainly composed of Proteobacteria, Bacteroidetes, Firmicutes, Fusobacteria, Cyanobacteria and other categories. The microflora in the intestinal tract of shrimps in each group were similar, but the proportions in each group were different to some extent. When fed for 14 days, the relative abundance of Proteobacteria in the 5% group was significantly lower than that in the 0% and 2% groups, and the relative abundance of Bacteroidetes and Firmicutes in the 5% group was significantly higher than that in the 0% and 2% groups (Fig. 10). Overall, feeding *E. faecium* can increase the number of beneficial bacteria and reduce the number of harmful bacteria at the phylum level of the intestinal flora.

## Discussion

Antioxidant systems play a vital role in protecting organisms from oxidative stress. As a key component of the shrimp defense mechanism, the antioxidant system helps maintain cellular homeostasis and ensures the overall health and survival of the species under various environmental conditions. In the cell metabolic process, oxygen free radicals are continuously updated, thereby maintaining homeostasis for organisms^29^. If free radicals are overproduced, it would result in lipid peroxidation. MDA is the main component of lipid peroxides with high toxicity of damaging cell structure and function. Its level indirectly reflects the activities of oxygen free radicals and lipid peroxidation in *vivo*, which in turn indicates the degree of cell damage^30^. The antioxidant enzyme system, composed of SOD and CAT, is responsible for scavenging the excessive free radicals and reducing lipid peroxidation damage. Therefore, it could accurately reflect the antioxidant status in *vivo* by monitoring the changes of SOD and MDA activities^31^. On the other hand, AOC is a comprehensive evaluation of antioxidant capacity. The higher T-AOC activity implies the enhancement of metabolism and immunity.

Research shows that fed *E. faecium* to mice with acute alcoholic liver injury found a significant increase in SOD and decrease in glutathione and MDA levels^32^. Another research reported that feed supplemented with 500 mg/kg *E. faecium* reduced serum MDA and enhanced the antioxidant capacity^33^. When Lactococcus lactis L19 and Enterococcus. faecalis W24 as feed additives at 1.0 × 10^8^ cfu/g, they could promote growth performance, enhance immune response and disease resistance of *Channa argus*, they also showed a protective effect on the fish intestine^34^. High-throughput sequencing revealed supplied L19 effectively enhanced the generation of probiotic bacteria and reduced the abundance of aquatic pathogens. Thus, adding *L. lactis* L19 had more effective protection than *E. faecalis* W24 or the mixture of them, whilst promoting digestive enzyme activity, antioxidant capacity, intestinal microbiota and morphology of *C. argus*^35^. These findings are consistent with the results of this study. In this study, the additive *E. faecium* caused an increase first, then decrease in MDA levels. This trend was also observed in SOD. They illustrated the higher metabolic rate and lipid peroxides in the shrimp fed with feeds supplemented with *E. faecium* after 14 d. In addition, SOD activity in the experimental group was significantly higher than that in the control*.* There was no significant difference in MDA content among the three groups. Furthermore, it was also shown that supplemented *E. faecium* could significantly improve the antioxidant capacity and immunity of *P. monodon*, meanwhile, significantly reduce the lipid peroxidative damage of *P. monodon*.

Studies have shown that a certain amount of antibiotics and *E. faecium* (SF68) is added to the basic feed of weaned piglets. The T-AOC levels in the liver, jejunum, and ileum of the *E. faecium* group were higher than those of the control and antibiotic groups. This proves that adding low-dose *E. faecium* SF68 to the feed reduces anti-oxidative stress in weaned piglets^36^. Our findings showed that T-AOC activity of *P. monodon* hepatopancreas peaked at 14th d. The T-AOC activity of experimental groups were higher than that of the control group. However, there was no significant difference between the control and *E. faecium* groups. At 28th d, the T-AOC activity in *P. monodon* hepatopancreas from 2 and 5% groups was significantly higher than in the control. It means that additive *E. faecium* improves the metabolism and immunity of *P. monodon*. In summary, using feed supplemented with *E.faecium* can significantly improve the antioxidant capacity of *P. monodon* and significantly reduce lipid peroxidation damage in *P. monodon*.

AKP and ACP are two important non-specific phosphohydrolases in the humoral immunity of crustaceans. They play an important role in eliminating pathogenic microorganisms in the immune response, as well as participate in various regulatory reactions. Hence, levels of AKP and ACP are often used to assess the immune status of crustaceans.ACP is a marker enzyme of lysosomes. In an acidic environment, ACP destroys foreign bodies with phosphate esters on the surface through hydrolysis to prevent infection^37^. Similarly, AKP is also an important component of mollusk lysosomal enzymes and participates in many reactions in crustaceans such as immune response, regulation of calcium and phosphorus metabolism, and keratin secretion in the body^38,39^. It has been proven that the commercial probiotic DBA® (Bifidobacterium sp, *Lactobacillus acidophilus* and *E. faecium*), prebiotics MOS and chitosan, synbiotics to promote Nile tilapia growth performance, innate immune system modulation and protection against *Aeromonas hydrophila*^40^. The effects of *E. faecium* of the host-associated bacterium isolated from adult Caspian roach intestine on growth performance, body composition, non-specific immunity and digestive enzymes activity of the fish^41^. The promoting effects of *E. faecium* on fish growth, boost imune system, and protection were effectively enhanced with continuous management^42^. These results are consistent with the results of this study.

The results of this study proved that AKP and ACP in *P. monodon* hepatopancreas from the three groups significantly increased. At 28th d, the AKP and ACP levels in the control group significantly decreased, whereas that of 2% and 5% groups had no significant change compared with that at 14th d. In summary, *E.faecium* supplemented feed can effectively enhance the activities of AKP and ACP in the hepatopancreas of* P. monodon*, and AKP and ACP can participate in the regulation of cellular processes such as phosphorylation and dephosphorylation. Changes in cellular phosphorylation status can affect the activity of proteins involved in immune responses and antioxidant defense mechanisms. This result is consistent with the results of SOD and T-AOC. *E.faecium* supplementary feed can effectively enhance the antioxidant and immune capabilities of *P.monodon* and enhance the immunity.

PO is an enzyme that widely occurs in invertebrates, playing a key role in nonspecific immunity^43^. The reported that in the defense system of shrimp, the recognition of foreign bodies, connection and enhancement of responses related to disease resistance are mainly realized by blood cells^44^. In this study, the PO activity of the control group showed minimal change at 0 d and 14 d, but it significantly increased at 28 d. The PO activity of 2% group significantly decreased during 14 d, then markedly increased at 28th day, and recovered to the level on 0 d. After 14 and 28 d feeding, the PO activity of 5% group was significantly lower than that on 0 d. The reported evidenced that PO activity showed no significant difference between sea cucumbers and the control group after feeding with yeast alone^45^. The PO activity of *Acidiopsis japonicus* was significantly higher than that of the control group only in the second week, and there was no significant difference between *A. japonicus* and control groups in the first, third, and fourth weeks. There was no significant difference in PO activity between the control group and *A. japonicus* fed with lactic acid bacteria^46^. They found that mixing 10^8^ CFU/g Lactobacillus with 0.2% isomaltose oligosaccharide increases PO activity in *P. vannamei* serum. These results are consistent with the results of this study. In this research, its impact on the immune and antioxidant systems of *P.monodon* may require further study.

TLR22 has the typical characteristics of the Toll family and belongs to type I family of transmembrane proteins. TLR22 plays an important role in the antiviral immunity of aquatic animals^46^. Studies have shown that viral nucleic acid analogues can upregulate *tlr* expression^47^. In this study, 5% *E. faecium* supplementation for 14 d caused a significant over-expression of *tlr22* in *P. monodon*, which suggested an enhanced immunity. Lysozyme is an important antimicrobial enzyme in blood that regulates the synthesis and secretion of other immune factors. It is also an important component of nonspecific immune system of crustaceans, playing an important role in immune response^48^. Supplementation of *Bacillus* and *Vibrio alginolyticus* compound bacteria in diets significantly up–regulated *lysozyme* expression in lymphocytes of *Litopenaeus vannamei*. This study also showed that *E. faecium* supplementation significantly increased the expression of *lysozyme* in *P. monodon* hepatopancreas. The IMD signaling pathway plays an antimicrobial role by producing antimicrobial peptides against gram-negative bacteria^49^. The IMD is also an adaptor protein containing the DEAD domain. When *imd* is mutated, the organism cannot produce antimyctide against the gram-negative bacteria. The Toll pathway activates nuclear transcription factors *dorsal* and *dif* through a series of signal cascades to produce antimicrobial peptides (AMPs) against gram-positive bacteria and fungi such as Crustin^50^. With the continuous development and improvement of molecular biology, studies on nuclear factor kappa-B (NF-κB) in aquatic invertebrates have been conducted extensively. NF-κB has been found in *Napus pinifera*, *Eriocheir sinensis*, *P. sinensis*, *P. monodon*, and *L. vannamei*, and its function has also been widely studied. When the organism is infected by gram-negative bacteria, the signaling cascade activates the NF-κB transcription factor *relish*, which enters the nucleus to promote the expression of antibacterial peptides and other immunity-related genes, and the PO cascade reaction also induces inflammatory factors such as iNOS that triggers an inflammatory response^51–53^. AMPs are key factors in the non-specific immune defense system. AMPs are bioactive peptides produced to defend against the invasion of external bacteria ^54^. Various AMPs have been reported in crustaceans such as Crustin in *L. vannamei, P. sinensis*, and *P. tritrucosa*^55,56^. It has been confirmed the *relish* was upregulated by infection with viruses and microorganisms^57^. In this work, the expressions of immunity-related genes in experimental groups increased first then decreased with the feeding time going on. It was hypothesized that the expression of immunity-related genes in hepatopancreas would not increase with the extension of feeding time, but down-regulate. Further studies would be thus warranted to explain this alteration. Our results only revealed that supplemented *E. faecium* significantly increased the expressions of immunity-related genes, i.e. *dorsal*, *crustin*, *imd* and *relish* in *P. monodon* hepatopancreas .Immune-related genes in *P.monodon* play a crucial role in coordinating immune responses. ROS produced by the antioxidant system can serve as signaling molecules in immune cells and affect the expression of immune-related genes. ROS-mediated signaling pathways may activate or repress certain genes, thereby promoting the overall immune response. Antioxidant enzymes, including SOD and T-AOC, may affect the expression of immune-related genes. These enzymes help regulate intracellular ROS levels, and their activity may influence redox-sensitive signaling pathways that control immune gene expression. The expression results of this immune-related gene are consistent with the results of SOD and T-AOC, indicating that the increase in the activity of antioxidant-related enzymes can significantly increase the expression of immune-related genes in the hepatopancreas of *P. monodon*, thereby enhancing the immunity of *P. monodon*. capacity and antioxidant capacity.

Vibrio is one of the common bacterial groups in the marine environment. This type of bacteria has strong adaptability and stress resistance, so it has become the dominant population in the seawater environment. Vibrio bacteria can cause vibrosis in shrimps, and it is still the most common and most harmful bacterial disease in the process of shrimp farming. When pathogens enter the shrimp body, they encounter the innate immune system and respond to them by releasing ROS through oxidative stress. Most cells have protective mechanisms to balance ROS production and avoid oxidative stress. Antioxidant-related enzymes can prevent the harmful effects of ROS and play a crucial role in protecting cells from oxidative stress and preventing or repairing oxidative damage. Studies have shown that after *Vibrio parahaemolyticus* attacks *P.monodon* for 6 h to 24 h, MDA levels in the hepatopancreas and gills are significantly increased compared with the control group, indicating that Vibrio can cause excessive formation of ROS and aggravate membrane Lipid peroxidation, leading to cell damage and oxidative stress^58^. The results of this experiment showed that both 2% and 5% *E. faecium* in the feed could reduce the average cumulative mortality of *P. monodon*, and the 5% group had the highest survival rate and the lowest cumulative mortality. It can be shown that adding *E.faecium* can improve the ability of *P.monodon* to resist bacterial infection. This is closely related to the fact that *E.faecium* can increase the expression of SOD, T-AOC, AKP, ACP and immune-related genes. The increase in SOD and T-AOC enzyme activities can more effectively deal with cell damage and oxidation stress response caused by excessive ROS. maintaining the balance of the antioxidant system, and high expression of AKP, ACP and immune-related genes can improve the immunity of *P.monodon*, enhance the disease resistance of *P.monodon*, and improve the survival rate.

Gut microbial structure and gut health are closely related, it is also closely related to the immune capacity and antioxidant system^59^. The richness index and diversity index are important indicators for evaluating the diversity and complexity of microorganisms. The differences in their values reflect the differences in community structure and the number of species, and their indicators are reflected by the number of effective OTUs divided. In this experiment, the number of effective OUTs in the 2% and 5% groups was significantly higher than that in the 0% group, indicating that the 2% and 5% groups had more microbial species than the 0% group. The Ace index and Chao1 index are commonly used to calculate the flora richness. The larger the two values, the greater the total number of species contained in the sample. Shannon index and Simpson index are used to calculate bacterial diversity, and the larger the two values, the higher the species diversity^60^. The results of this experiment showed no significant changes in Shannon index and Simpson index values, indicating that the species richness of the gut microbiota was altered but its diversity was unchanged. From the perspective of phylum level, in this study, Proteobacteria, Bacteroidetes and Firmicutes were the dominant flora in the intestinal tract of *P. monodon*, and the proportion of phyla among each group existed. This indicated that the changes in the microbiota in the *P. monodon* gut were affected to a certain extent by the culture environment, culture cycle and individual health issues. This experimental study shows that the relative abundance of Proteobacteria in the intestinal tract of *P. monodon* in the 2% and 5% groups is lower than 0%.In summary, it shows that *E.faecium* can inhibit the reproduction of some harmful bacteria in the intestine. Guide and improve gut health. This is consistent with the results that *E.faecium* can enhance the activities of SOD, T-AOC, AKP, and ACP enzymes, increase the expression of immune-related genes, and increase resistance to bacterial infections. Gut microbes may also influence the expression and activity of host antioxidant enzymes, such as SOD. Microbial products or metabolites can signal the host's antioxidant defense system and reduce oxidative stress. A diverse and balanced intestinal microbiota contributes to immune regulation, affecting the activity of immune cells and the expression of immune-related genes. Gut microbes contribute to the production of metabolites such as short-chain fatty acids (SCFA) with immunomodulatory and antioxidant effects. It is also possible that these metabolites can be produced by microbial fermentation processes in the gut to produce bioactive compounds that may have immune and antioxidant effects and influence the host's immune function and antioxidant defense, contributing to the overall health of *P. monodon.*

## Materials and methods

### Ethics statement

*P. monodon* used in this study came from the industrialized artificial breeding. All experimental procedures were strictly performed following the relevant national guidelines of China, e.g. “Reporting of In vivo Experiments (ARRIVE) guidelines” and “Guidelines for Experimental Animals” from the Ministry of Science and Technology (Beijing, China). Further, the Institutional Animal Care and Use Ethics Committee of Tianjin Agricultural University had approved our study. All efforts were made to minimize the suffering of sampled fish individuals. National Standard of the People's Republic of China: Laboratory animal—Guideline for ethical review of animal welfare.the ethical code: GB/T 35892-2018.the date of approval: 2018-02-06.

### Test animals and feed preparation

In this study, *P. monodon* were purchased from South China Sea Fisheries Research Institute (Guangdong, Guangzhou, China). The initial average body length of *P. monodon* was 6.00 ± 0.82 cm, and the initial average body weight was 1.54 ± 0.67 g. The basic baits of *P. monodon* were purchased from Tongwei Agricultural Development Co., Ldt(Chengdu, China). Their nutrient contents were lysine ≥ 1.8%, crude protein ≥ 40%, total phosphorus ≥ 4%, crude fiber ≤ 6%, and moisture ≤ 12%, respectively. *E. faecium* were cultivated in the laboratory (Genbank accession no. MF928076; number of viable bacteria: 10^9^ cfu/L), LB medium was used for the activation of *E. faecium*, the compositions of LB medium were Tryptone (10 g/L), yeast extract (5 g/L), NaCl (10 g/L).

### Test groups and sample collection

The *E. faecium* was mixed into 480 g baits at 0%, 2% and 5% ratio everyday. The required weight of the basal diet was spread in a single layer on a pallet each day, and then the bacterial suspension was sprayed uniformly on the pellets with a fine-mist sprayer to achieve the required amount of bacteria for each group of diets. At the same time, the same volume of sterile saline was sprayed on the control diets. Finally, soybean oil (at 1% by weight of the basal diet) was sprayed on all diets. The pellets were allowed to dry at room temperature and then sealed in bags and stored at 4 °C. Experimental *P. monodon* were randomly divided into three groups. Each group consisted of three replicates, there were a total of 500 *P. monodon* individuals for each replicate in a container (100 cm × 100 cm × 100 cm). After mixing, the feeding diets were dried, then fed to *P. monodon* six times per day (i.e. at 5:00, 9:00, 13:00, 17:00, 21:00, and 1:00). The total daily feeding amount was 1% of the total body weight of shrimps. With a feeding cycle at about 28 days, water temperature was controlled in 25 ~ 33 °C, and salinity was 25‰. The samples were collected at 0 d, 14 d, and 28 d of the culturing period, a total of 40 shrimp were taken from each group. After sampling, the shrimp were immediately sent to the laboratory for collection of hepatopancreas, which were cryo-preserved at − 80 °C until use.

### Enzyme activity determination

The tissues were thawed before measurement. The tissues were weighed and diluted 10 times with normal saline, ground with an electric homogenizer (1000 rpm), the homogenate was poured into a centrifuge tube, which was then centrifuged at 5500 rpm for 30 min at 4 °C, then the supernatant was collected determination of enzyme activity. ACP was determined by spectrophotometry, visible light colorimetry for AKP, TBA method for MDA activity, colorimetry for T-AOC, and double antibody sandwich method for SOD and PO activity.

### Quantitative real-time PCR analysis

The tissues were homogenized by grinding apparatus to extract total RNA using RNAiso reagent (Takara Biomedical Technology Co., Ltd., Shuzo, Japan) according to the manufacturer's instructions. The total RNA was then dissolved in 50 µL RNase-free water. The quality of total RNA was checked with the agarose gel and concentration were measured by NanoDrop 2000 (Thermo Fisher Scientific Co., Ltd., Waltham, Massachusetts, USA). The PrimeScript RT reagent Kit with gDNA Eraser (Takara Biomedical Technology Co., Ltd., Shuzo, Japan) was used to reverse-transcribe the first strand cDNA from the total RNA.

Primers were designed using *P. monodon* sequences of *tlr22*, *dorsal, lysozyme, crustin, imd, relish* in NCBI and primer design software PrimerPremier5.0 The list of primers is shown in Table 2. EF-1α was employed as internal reference, with primers designed as described elsewhere. When the internal reference gene and the target gene were amplified, the same amount of template was added, and after amplification, melting curve analysis was performed to determine non-specific amplification.

**Table 2:** Primers sequence used for PCR.

Take things	Sequence (5 '-3')
TLR22-F	GCTGAACGATAACCCCTTGTG
TLR22-R	GATAAAGCCTGGTGACATTACTG
Dorsal-F	GATGGAATGATAGAATGGGAAGC
Dorsal-R	CACTGGTACTCTTGTCTGGTGGTC
Lysozyme-F	TGTTCC GAT CTG ATGTCC
Lysozyme-R	GCT GTT GTA AGCCACCC
Crustin-F	AATGGCTCGTCTTTGTGTCTT
Crustin-R	CTTTCCACGGGTTGCTTAGGT
IMD-F	CGACCACATTCTCCTCCTCCC
IMD-R	TTCAGTGCATCCACGTCCCTC
Relish-F	CTACATTCTGCCCTTGACTCTGG
Relish-R	GGCTGGCAAGTCGTTCTCG

Expression patterns of these genes were analyzed using quantitative real-time PCR (qRT-PCR). The qRT-PCR assay was performed using SYBR Premix Ex TaqII (Takara Biomedical Technology Co., Ltd., Dalian, China) on a Bio-Rad iQ5 machine (Bio-Rad Laboratories Co., Ltd., Hercules, California, USA). The qRT-PCR conditions were as follows: denaturation at 95 ℃ for 30 s, followed by 40 cycles of 95 ℃ for 5 s, annealing at 60 ℃ for 30 s. The relative quantification of the target and reference genes was evaluated according to standard curves. Each experiment was conducted in triplicate. The relative stability measures (M) of the reference genes were calculated by calculation formula.

### Challenge experiment

The *Vibrio parahemolyticus* strains used in this experiment were purified and cultured in our laboratory and stored at – 80 ℃. The 300 mL of the activated *V. parahaemolyticus* test bacterial solution at a concentration of 10^8^ CFU/mL (LD50) was added to 20 L water. After the 28-day separated feeding, 10 shrimp were randomly selected from each group for the challenge test. There were three parallel in each group. The feeding was stopped for 1 day before the *V. parahaemolyticus* challenge test, then the experimental *P. monodon* were isolated and cultured in the tank. After soaking overnight, the *P. monodon* were taken out, the water with specific bacterial concentration was completely changed. *P. monodon* were temporarily cultured. The morbidity and mortality of the infected *P. monodon* was observed everyday. The test period was 7-day. The average cumulative mortality was calculated.

### Analysis of the structure of intestinal flora

The gut microbiota structure was analyzed by high-throughput sequencing by Beijing Biomaike Biotechnology Co., Ltd (Beijing, China). The effects of *E. faecium* (R8a) on the intestinal tract of *P. monodon* were analyzed from three aspects: statistics of operational taxonomic units (OTUs), species diversity analysis and intestinal flora structure in the shrimp intestine.

### Data processing and statistical analysis

The relative expression of the target genes was calculated using the 2^-△△Ct^ method. All data in this experiment were processed by Excel 2019 and SPSS18.0 for ANOVA. *P* < 0.05 indicates a significant difference, and *P* < 0.01 indicates a very significant difference. All of the data are expressed as mean ± standard error (n = 3).

## Conclusions

The results prove that E. faecium feed supplementation increases antioxidant and non-specific immunity activity in the hepatopancreas of *P. monodon* and reduces lipid peroxidation, enhance disease resistance, increase the expression of immune related genes. Based on these findings, we suggested that 5% E. faecium feed supplementation is optimal.

## Data Availability

All of the data used in this study will be shared by the corresponding author upon request.
